# Olive Pomace as a Reinforcing Agent in PETG Filaments for 3D Printing in the Context of Circular Economy

**DOI:** 10.1007/s42824-025-00186-5

**Published:** 2025-09-11

**Authors:** N. Sánchez-Ávila, M. Carmona-Cabello, M. Cano-Galey, P. E. Romero, M. P. Dorado

**Affiliations:** 1 Dept. of Physical Chemistry and Applied Thermodynamics, Campus de Rabanales, Universidad de Córdoba, Campus de Excelencia Internacional ceiA3, Córdoba, Spain; 2 Dept. of Mechanical Engineering, School of Engineering, University of Birmingham, Birmingham, B15 2TT UK; 3 Andaltec, Plastic Technology Center, Martos, Jaén Spain; 4 Dept. of Mechanical Engineering, Campus de Rabanales, Universidad de Córdoba, Campus de Excelencia Internacional ceiA3, Córdoba, Spain

**Keywords:** Biomass valorization, Plastic composite, Eco-friendly filler, Material extrusion, Mechanical properties, Materials characterization

## Abstract

3D printing is increasingly present in many industrial applications, where petroleum-based polyethylene terephthalate glycol (PETG) is gaining importance. This is due to both its mechanical properties and ease of printing. In this context, with the aim of reducing the presence of plastics during the manufacturing process, the addition of olive pomace (OP) as an additive is proposed. OP is a residue from the olive oil industry that represents an environmental challenge, due to its high content of organic matter and phytotoxic compounds. To optimize the fabrication and further analysis of 3D printing filaments composed of a mixture of PETG and OP, a design of experiment (DoE) was used. To gain knowledge about the relationship between mechanical properties of different OP/PETG blends, OP particle size as filler, and extrusion number to produce the optimal filament, analysis of variance (ANOVA) besides response surface methodology (RSM) was applied. Additionally, thermogravimetric (TGA), differential scanning calorimetry (DSC), infrared (IR) spectroscopy analysis, and scanning electron microscope (SEM) of the new composite material blends were carried out. Subsequently, different OP/PETG filaments were produced via material extrusion additive manufacturing. This study revealed that the addition of 8% (v/v), < 100 µm OP particle size, fabricated using a double extrusion process, to PETG composites exhibited significantly enhanced mechanical properties. In particular, the incorporation of OP filler resulted in a remarkable increase in yield strength (35%), tensile strength (8.4%), and Young’s modulus (27%). Furthermore, slight improvement in ductility, evidenced by an increase in elongation at yield (4.18%) and at break (5.16%), demonstrates the potential of OP as a valuable and sustainable reinforcement material for PETG composites. These findings pave the way for the development of high-performance environmentally friendly materials derived from residues, in the context of circular economies, for applications that require stiffer, stronger, and more tenacious material than straight PETG.

## Introduction

To automate manufacturing processes while reducing costs and waste, implementing digital transformation and moving towards Industry 4.0 is key. This is leading to new and more sustainable manufacturing technologies, with a particular focus on reducing carbon footprint (Camilleri 2019).

One of the most innovative and currently booming methods to produce industrial parts, with excellent results, is additive manufacturing. This emerging technology allows transforming a digital model into a solid object without the need for expensive tools, modules, or processes (Attaran 2017; Dass and Moridi 2019).

The most widespread 3D printing technique is based on thermoplastic material extrusion (MEX), following standard ISO 52900. The physics of the process is simple. An extruder melts a thermoplastic filament and deposits it, layer by layer, until the desired component is produced. This technique allows the production, in a single low-cost step, of small and medium batches of parts with complex geometry (Shanmugam et al. 2020). Moreover, this technology is characterized by considerable material savings, with minimal waste (Szykiedans et al. 2017; Wickramasinghe et al. 2020).

Another advantage of MEX is the wide range of materials available on the market. Among them are polylactic acid (PLA), acrylonitrile butadiene styrene (ABS), or polyethylene terephthalate glycol (PETG) (Park et al. 2022; Durga Rajesh et al. 2023). PLA is a biodegradable material widely used in 3D printing, as it is produced from renewable organic resources, i.e., sugar cane or corn starch. Due to its high surface energy, PLA prints easily without releasing harmful vapors during the extrusion process. Its major fields of application are the medical and food sectors, where it is widely used. However, PLA shows low deflection temperature (60 °C) and poorer mechanical properties than other thermoplastics. In addition, in contact with water, it decomposes by hydrolysis and its resistance to ultraviolet radiation is limited. Due to these handicaps, PLA is hardly used in industrial applications.

Also, ABS is a thermoplastic widely used in MEX, as it exhibits excellent mechanical properties and good impact resistance. However, it releases noxious fumes when it melts and generates warping during printing. These ABS weaknesses have led PETG to gain importance in additive manufacturing using MEX.

PETG exhibits medium-to-high mechanical properties in terms of toughness, ductility, transparency, and impact resistance. Its deflection temperature is 70 °C. It shows high chemical resistance, does not react with water, fungi and mold, and resists UV exposure (Petousis et al. 2023). In the broader context of sustainable additive manufacturing, PETG has gained significant attention for its excellent printability and mechanical properties in MEX 3D printing. PETG filament prints effortlessly, has excellent adhesion between layers, and does not release harmful fumes during printing. Due to its high chemical and thermal stability, besides fast extrudability, it is particularly safe and suitable for the food industry. This material is frequently used for tooling and spare part manufacture in engineering applications, even under low and high strain rate loading conditions (Vidakis et al. 2020). This material is based on polyethylene terephthalate (PET); unlike PET, PETG offers the advantage of being recyclable multiple times without significant loss of mechanical properties (Vidakis et al. 2021). Furthermore, for the development of PETG composites for MEX, various types of fillers, ranging from ceramics and inorganic additives (i.e., recycled fine powder glass (Petousis et al. 2024a), antimony tin oxide (Petousis et al. 2024b) and silicon nitride (Michailidis et al. 2024)) to other nature-sourced additives (i.e., biochar (Bolanakis et al. 2025)), have been explored. These investigations underscore the versatility of PETG as a matrix and the growing interest in eco-friendly composite solutions.

As they are composed of high starch, protein, and fiber (cellulose, hemicellulose, and lignin) content, biomass can impart different properties to the resulting composites. On one hand, cellulose, the most abundant component in biomass, improves thermoplastic material mechanical properties. For this reason, it has been used to reinforce polymers, improving their strength and stiffness (Abdul Khalil et al. 2017; Wang et al. 2018; Dahiya et al. 2020; Jaiswal et al. 2022; Sharma et al. 2023). On the other hand, although less studied, hemicellulose also shows interesting potential for improving mechanical properties. Its incorporation in plastic materials has shown flexibility and toughness increase of printed objects. As for lignin, its inclusion in plastic materials can contribute to higher impact resistance and reduced brittleness, thus improving overall toughness of printed products (J. Yang et al. 2019a, b; Tanase-Opedal et al. 2019; Ebers et al. 2021; Agustiany et al. 2022; Zhang et al. 2022).

In Spain, the olive oil industry is of great importance within the food industry, due to its leading position in world olive oil production. This sector plays a crucial role in the country economy, generating employment and significantly contributing to the national gross domestic product (GDP). However, olive oil production also brings environmental challenges, i.e., the management of olive pomace (OP). This is a by-product generated during the mechanical extraction of olive, in the two-stage olive oil production process. It is the solid fraction (with high content of organic matter and phytotoxic compounds) remaining after the pressing or centrifugation of olives to separate the oil (Koutrotsios et al. 2016; Bajoub et al. 2018). This residue is primarily composed of olive skins, pulp, stone fragments, residual water, and oil. The composition and properties of OP can vary depending on the olive oil extraction method.

The inappropriate use or accumulation of OP may cause environmental problems, i.e., soil and water pollution, negatively affecting local ecosystems. Several management strategies are currently available to reduce OP environmental impact. Among them, composting, fuel for boilers, and biogas production through anaerobic digestion are the most widely used recovery routes (Messineo et al. 2020; Bouhia et al. 2023). However, to address the environmental problem while optimizing the use of resources in the olive oil industry, the promotion of more sustainable technologies for OP management is essential (Bouhia et al. 2023).

OP contains high percentages of lignin, cellulose, and hemicellulose. For this reason, its use in various polymer matrices as a reinforcing agent and eco-friendly filler has been researched. Oulidi et al. (2022) developed a polyamide 6 (PA6)/OP powder biocomposite using in situ polymerization, with OP contents ranging from 5 to 20% (w/w). Non-chemically treated OP exhibited particle sizes of 160–250 µm. Structural, thermal, and morphological analyses showed that up to 20% (w/w) OP did not significantly alter PA6 properties, suggesting its potential to reduce production costs while valorizing agricultural waste. In turn, Kawano et al. (2023) investigated the use of OP and marble powder as fillers in polypropylene (PP) composites, highlighting their potential to reduce environmental impact of these industrial residues. Through mechanical grinding and ionic liquid treatment, OP performance as a reinforcing agent was enhanced. Optimized formulations showed significant improvements in mechanical strength and Young’s modulus, confirming their suitability as sustainable materials within the framework of circular economy. Also, Aljnaid and Banat (2021) studied the effect of OP flour addition on mechanical, water uptake, morphological, and thermal properties of PP composites with and without coupling agents. The use of coupling agents significantly improved impact strength, tensile, and flexural properties compared to OP/PP composites without coupling agents. Thermal analysis revealed a decrease in melting enthalpy, crystallization enthalpy, and crystallinity percentage upon filler and coupling agent incorporation. These findings demonstrate that OP can be a viable reinforcement for PP, providing enhanced mechanical and morphological performance when combined with suitable coupling agent proportions. Furthermore, Kaya et al. (2018) tested the use of OP as filler in PP composites. OP was incorporated into PP at weight fractions ranging from 10 to 40% (w/w) using a high-speed thermo kinetic mixer. The effects of OP content on mechanical, viscoelastic, thermal, chemical, crystallographic, and morphological properties were evaluated. At 40% (w/w) OP loading, Young’s modulus and flexural modulus increased by approximately 62.5% and 19%, respectively. Additionally, storage modulus and thermal stability improved significantly with higher OP content. Mousa et al. (2009) focused on incorporating OP into toughened polyvinyl chloride (PVC) blends with OP content ranging from 0 to 30% (w/w). The blends were evaluated for processability, flexural behavior, dynamic mechanical properties, thermal stability, and morphology, via SEM. Attenuated infrared spectroscopy indicated proton donor–acceptor interactions between OP and PVC chains, likely involving hydrogen or chlorine atoms of PVC and hydroxyl groups of lignin, which contributed to composite improved properties. On the other hand, Banat et al. (2022) studied the influence of varying weight fractions (0–40%) of OP in linear low-density polyethylene (LLDPE) composites, with and without coupling agent addition (0%, 5%, and 10% (w/w)). Composites were produced via extrusion and hot pressing. Tensile strength at yield increased up to 20% with OP loadings up to 20% (w/w), with a slight enhancement when coupling agents were used. Elastic modulus significantly improved, reaching over 800 MPa with coupling agent addition, although a reduction to 550 MPa was observed at 40% (w/w) filler content. Impact strength decreased with increasing OP content, but doubled with 10% (w/w) coupling agent in 10–40% (w/w) loading range. The study confirms the compatibility of OP as a bio-based filler in LLDPE composites, supporting its potential application in sustainable biocomposite materials.

To date, no previous studies have shown interest on the use of OP-based composite materials for MEX additive manufacturing, i.e., the use of OP as reinforcing agent in 3D printing filaments. Furthermore, no research investigating the thermomechanical properties of a composite composed of OP and PETG (one of the most common materials in MEX processes) has been identified. This proposal introduces a significant advancement in PETG composites for 3D printing by including OP, a widely available agricultural waste, as a sustainable natural filler. While prior research (Petousis et al. 2024a, 2024b; Michailidis et al. 2024; Bolanakis et al. 2025) explored various fillers, from recycled fine powder glass (RFPG) to ceramics and biochar, primarily targeting specific mechanical, electrical, or thermal enhancements, the present approach offers unique advantages. Unlike synthetic or other industrial residue-derived fillers, OP provides waste valorization from the olive oil industry. This is coupled with the potential for natural coloration and comparable reinforcement effects in terms of increasing stiffness and strength. To the best of our knowledge, this is the first time that OP is explored as PETG filament filler for MEX. In the present work, its potential as cost-effective, environmentally friendly alternative to conventional fillers are demonstrated. Furthermore, particle size optimization and process control (e.g., double extrusion) offer crucial insights into effectively incorporating OP bio-based fillers into PETG matrices for additive manufacturing. This approach strongly supports circular economy principles through environmental sustainability and waste valorization, leading to the development of high-performance, eco-friendly materials for industrial applications. To design the experiments, a Box-Behnken response surface methodology was used. Fifteen distinct filament formulations, using a screw extruder, each incorporating varying OP contents (1%, 8%, 15% v/v) and particle sizes (ranging < 100 µm, 100–150 µm, 150–200 µm) undergoing one, two, three extrusion cycles, were fabricated. These filaments were subsequently used to produce standardized specimens via MEX, which were mechanically characterized through tensile testing.

## Methodology

### Origin and Preprocessing of Olive Pomace Powder

**Fig. 1:**
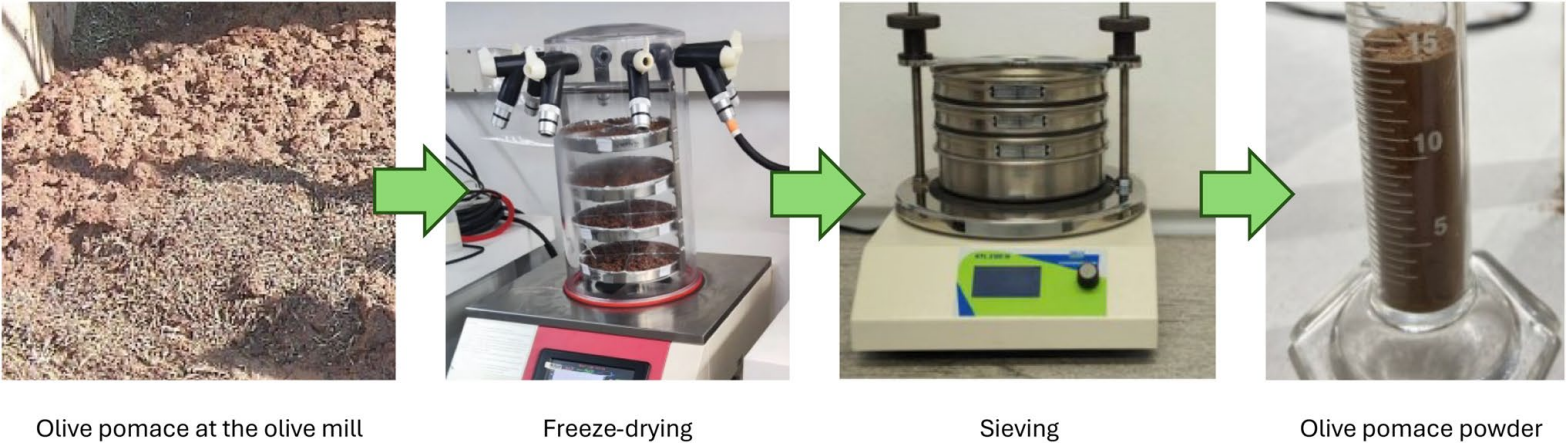
Preparation of olive pomace powder

OP was supplied by “San Francisco” olive mill, located in Baena (Córdoba, Spain). It was provided after the most widely adopted process in olive oil extraction, that is, two-phase centrifugation procedure. To remove its initial moisture, OP was lyophilized 48 h. Subsequently, OP powder (Fig. 1) was sieved into the following specific fractions: 150–200 µm (particles that passed through a 200-µm sieve but were retained on a 150-µm sieve); 100–150 µm (particles that passed through a 150-µm sieve but were retained on a 100-µm sieve); and < 100 µm (particles that passed through a 100-µm sieve). This choice aligns with the most common particle sizes investigated in similar composite studies. Specific size ranges were selected based on the capabilities of our milling and sieving process, alongside considerations for achieving adequate dispersion within the polymer matrix. This particle size range has also been successfully used previously by Oulidi et al. (2022) and is specifically recommended by companies specialized in 3D printing filament manufacturing.

### Raw Material Preparation and Characterization

PETG pellets were supplied by Smart Materials (Alcalá la Real, Spain). Previous to processing, pellets were introduced in an oven for 2 h at 60 °C. Once the moisture was removed from the material, PETG pellets were ground and sieved at 1 mm. Next, the pellets and OP were mixed and homogenized in the desired proportions. Pure PETG was used as a reference for the subsequent tests.

A comprehensive physicochemical characterization of the samples was conducted using a combination of proximate, ultimate, and compositional analyses. Proximate analysis focused on determining sample basic components by standardized methods. Moisture, ash, and volatile matter content were determined according to EN 14774, EN 14775, and ASTM D3173-03, respectively. Fixed carbon (FC) content was calculated by subtracting, to the initial mass, the sum of moisture, ash, and volatile matter. It represents the solid residue that remains after the sample has released the volatile elements. Ultimate analysis aimed to identify sample elemental composition, including C, H, N, S, and O. A Leco series 928 elemental macroanalyzer (LECO Corporation, USA) was used. The oxygen content was calculated by subtracting the sum of all other elements from 100% (dry basis). Compositional analysis focused on quantifying the specific constituents of the biomass, namely hemicellulose, cellulose, and lignin. For this analysis, the protocol outlined in NREL/TP-510–42618 was followed. To ensure the reliability of these analytical results, analyses were performed in triplicate.

### Design of Experiment

The experimental design was developed following the Box-Behnken methodology, a type of response surface methodology (RSM). With the aim of optimizing a specific response, RSM was chosen for this study due to its efficiency in modeling and analyzing problems influenced by multiple variables. This method is particularly effective for fitting second-order (quadratic) models without requiring testing all possible combinations of factor levels. Unlike full factorial designs, the Box-Behnken design requires fewer experimental runs, making it more efficient and cost-effective. Additionally, it prevents extreme (corner) points in the design space, which can be advantageous when such conditions are impractical or potentially damaging to the system.

The Box-Behnken experimental design was developed using Statgraphics Centurion 19 software. Three critical parameters were investigated, namely OP/PETG ratio, OP particle size, and extrusion cycles (Table 1 shows the 15 runs that comprise the DoE):

- As most authors focus on the influence of OP content incorporated into thermoplastic matrix, to determine the maximum OP content that could be added to PETG (still allowing the filament to be successfully produced using a extruder), sensitivity tests were conducted. Additionally, it was verified that the resulting filament possessed the necessary qualities to be 3D printed without issues.
- As previously mentioned, particle size was selected within the range commonly used by other authors when incorporating OP into a thermoplastic matrix. Furthermore, sieve sizes of 100, 150, and 200 µm correspond to the standardized sieve sizes commonly available in laboratories. Sieving becomes more challenging as the mesh size decreases. This fact increases the time required for this stage and consequently raises the final cost of the filament. The inclusion of this factor intended to assess its significance and determine whether larger particle sizes can be used without negatively affecting the filament mechanical properties.
- To ensure mixture homogeneity, manufacturers of 3D printer composite filaments composed of multiple materials, typically perform several extrusion cycles. This procedure has a direct impact on the final cost of the filament, as it doubles or triples the extrusion expenses. Therefore, to identify the influence of particle dispersion on mechanical properties of printed specimens, this parameter was included.

**Table 1:** Design of experiments for filament optimization

Experiment	% OP (v/v)	Particle size (µm)	Extrusions number	Nomenclature
1	8	200	1	8% OP/PETG 200–1
2	15	200	2	15% OP/PETG 200–2
3	1	150	3	1% OP/PETG 150–3
4	15	150	3	15% OP/PETG 150–3
5	8	100	1	8% OP/PETG 100–1
6	8	150	2	8% OP/PETG 150–2
7	1	100	2	1% OP/PETG 100–2
8	1	200	2	1% OP/PETG 200–2
9	15	100	2	15% OP/PETG 200–1
10	15	150	1	15% OP/PETG 150–1
11	1	150	1	1% OP/PETG 150–1
12	8	150	2	8% OP/PETG 150–2
13	8	100	3	8% OP/PETG 100–3
14	8	150	2	8% OP/PETG 150–2
15	8	200	3	8% OP/PETG 200–3
Blank	0	1000	1	0% OP/PETG 1000–1

*OP*, olive pomace; *v*, volume

### Filament Manufacturing

OP/PETG composite filaments were prepared using a melt blending technique. The extrusion process was conducted using a Filabot EX2 extruder (Filabot, USA), equipped with a three-stage compact extrusion screw, which includes distinct feeding, compression, and extrusion phases, a cooling unit and a winding unit.

In the feeding phase, the polymer mixture (PETG pellets and OP particles) was introduced into the extruder hopper, where the volumetric screw pushed the material forward. The amount of polymer fed depended on the pellet apparent density and the screw rotation speed. As the screw rotated, it captured and advanced the material towards the barrel opening. The study incorporated variations in OP particle size, according to DoE, keeping PETG pellet size constant at one mm. These variations, along with the differing densities of the two materials, were expected to influence the homogenization process, altering shear forces and viscosities throughout the extrusion phases. Extrusion parameters, such as temperature (200 °C), screw speed, and feed rate, were carefully adjusted to produce uniform filaments with a target diameter of 1.75 mm. Throughout the melt extrusion course, the filament diameter was constantly monitored using a digital caliper (Brammer, Coslada, Spain), which allowed us to ensure it remained within the target range of 1.75 ± 0.5 mm. Once extruded, the molten filament was cooled using fans to solidify it before being wound. For the second and third extrusion cycles, the filament from the previous cycle was crushed with a grinder and reintroduced into the extruder. This iterative process ensured consistency and recyclability in filament production.

### Test Specimen Printing

**Fig. 2:**
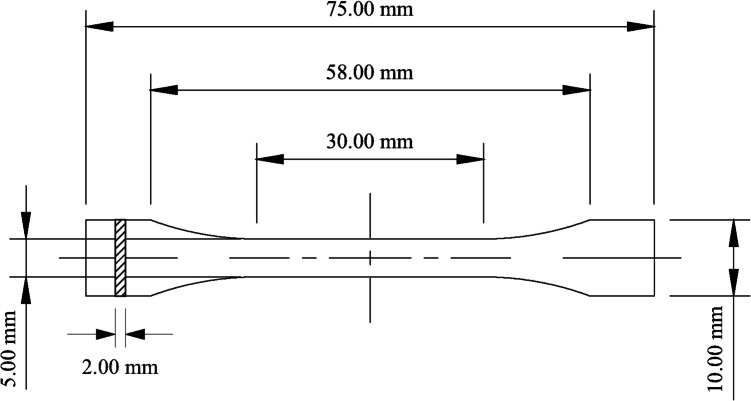
Technical specifications for ISO 527–02 Type 1BA dumbbell tensile test specimen

Once filaments were produced, tensile test specimens were designed and, subsequently, manufactured according to ISO 527–02 (Fig. 2) using MEX. Selected sample size was 75 × 10 × 2 mm^3^. For each experiment, ten specimens were printed.

For the manufacturing process, a Bowden 3D printer, Creality model Ender 3 (China), with a nozzle diameter of 0.6 mm and a printing volume of 220 × 220 × 250 mm^3^ was used. Printing temperature, speed, layer thickness, and infill density significantly impact performance and characteristics of 3D printed parts (Grasso et al. 2018). The selection of both filament extrusion and 3D printing parameters was guided by a combination of established industry best practices, specific material specifications, and extensive preliminary experimentation. These preliminary tests were vital to identify robust processing conditions and confirm that all material compositions defined by the DoE could be consistently extruded and successfully 3D printed. Table 2 summarizes MEX technique parameters for printing OP/PETG composite specimens. To achieve optimal printing results for OP/PETG composite filaments, based on both preliminary experiments and recommendations from the printer manufacturer, parameters were selected.

**Table 2:** MEX technique parameters for printing OP/PETG composite specimens

Parameter	Value
Nozzle diameter (μm)	600
Printing speed (mm/s)	50
Infill pattern (linear)	0º/45º
Layer height (μm)	200
Infill density (%)	100
Nozzle temperature (ºC)	200
Bed temperature (ºC)	70

*MEX*, material extrusion additive manufacturing; *PETG*, poly (ethylene terephthalate) glycol; *OP*, olive pomace

### Mechanical Characterization

To measure mechanical properties, once specimens were manufactured according to DoE, tensile tests were performed. Tests were carried out in accordance with ISO 527–2:2012 standard for conditioning and testing. They were performed using an Instron®’s model 3382 R&N 100 kN metal testing packages (Instron, Spain). Failure strain, Young’s modulus, and tensile strength were calculated. Each specimen underwent testing ten times and the resultant average values were reported.

Resulting responses were extracted from the test curves. Besides, a three-way analysis of variance (ANOVA) with interaction was performed. ANOVA enables the simultaneous comparison of more than two groups to assess the presence of a relationship among them. Results are considered statistically significant when *P* < 0.05. This statistical analysis yielded a behavioral equation, elucidating the main effects of each parameter on mechanical properties and their interactions. RSM approach together with ANOVA allowed analyzing the overall mechanical performance of OP/PETG filaments.

Following specimen fabrication (ten replicates per experiment), mechanical testing was conducted. To both assess mechanical properties of 15 OP/PETG composites and identify the optimal filament composition, failure strain, Young’s modulus, and tensile strength were measured. Table 1 shows samples categorized according to DoE. Controlling variables were OP/PETG ratio, OP particle size, and extrusion number. Based on preliminary experiments, to investigate 3D printability of OP/PETG filaments, the highest OP content in OP/PETG blends was selected (Osman and Atia 2018). Due to the high blend viscosity and thermal instability, it was found that 3D OP/PETG filaments with OP content above 15% (v/v) could not be 3D-printed. For this reason, PETG with 1%, 8%, and 15% OP (v/v) were the studied mixtures.

### Thermal Properties Analysis

The thermal degradation behavior of OP/PETG composites was explored by using a thermogravimetric analyzer differential scanning calorimetry, TGA DSC 3 + (Mettler Toledo, Spain). Each filament, positioned in ceramic crucibles, was tested under 50 mL min^−1^ N_2_ flow from room temperature to 873 K, with a heating rate of 3 K min^−1^. To ensure experimental reproducibility, each experiment was conducted in triplicate and results were averaged. Experimental results showed reproducibility within the range of ± 3%. For each test, a blank run was conducted to compensate buoyancy effects, using an empty pan.

For DSC analysis, a DSC Q200 calorimeter equipped with a Standard Cell FC module (TA Instruments, version 2.0, New Castle, DE, USA) was used. The system operated in standard mode and used a refrigerated cooling system. DSC experiments (Run 1, RunSerial 6760) were conducted under high-purity nitrogen atmosphere. The software version was V24.11 Build 124.

### Chemical Structure Analysis

Sample chemical structure was analyzed using Fourier-transform infrared spectroscopy (FTIR) with an ALPHA spectrometer (Bruker, Germany). It was equipped with an attenuated total reflectance (ATR) module. The ATR crystal used was diamond. Spectra were recorded over a wavenumber range from 4000 to 400 cm^−1^, with a spectral resolution of 4 cm^−1^. Data were collected at 1 cm^−1^ intervals. For each sample, ten replicates were provided. Spectral data were exported and analyzed using Unscrambler X software version 10.5 (Camo Analytics, USA). To prevent the scatter phenomenon or diffuse radiation, the first two treatments applied were standard normal variate (SNV) and detrending (DT). Next, a spectral signal smoothing using Savitzky-Golay (SG) derivative method was performed. Finally, to study differences and similarities between samples, focusing on sample clustering, a principal component analysis (PCA) was carried out.

### Scanning Electron Microscope Analysis

The surface and fracture cross-sections of the OP/PETG composite tensile samples were examined using a scanning electron microscope (SEM). This helped to assess the structural and morphological characteristics of the specimens, providing insights relevant to additive manufacturing. For this analysis, a SEM Leica AC600 high vacuum coater (Barcelona, Spain) was used. Samples were placed on a Cu/Zn support by carbon paint and coated in 10 nm gold. Eventually, prior to the microscopic analysis, samples were kept in an oven at 35 °C.

## Results and Discussion

### Raw Materials Physico-Chemical Characterization

To further the knowledge of the material properties, an exhaustive physicochemical characterization of the samples was carried out, incorporating proximate, final, and compositional analyses. Results are summarized in Table 3. Experimental error was kept within ± 3% of the mean values. The composition of the raw material is vital for understanding three fundamental aspects: its density, behavior at elevated temperatures, and thermal stability. The amount of lignocellulosic material in the blend are relatively low, considering that the olive pomace (OP) content does not exceed 15% (v/v), which translates to less than 9% (w/w). During processing, exposure to extrusion temperatures exceeding 195 °C and printing temperatures above 240 °C can lead to thermal decomposition. This decomposition releases volatile compounds, potentially creating voids in the filament structure and making it challenging to maintain a uniform filament diameter (Carmona-Cabello et al. 2025).

**Table 3:** Physico-chemical characterization of PETG and OP biomass

	Proximate analysis (w/w, %)				**Ultimate analysis (w/w, %)**						Compositional analysis (w/w, %)		
Sample	Moisture	Volatiles	Ash	FC	**C**	H	N	S	O	H/C	Cellulose	Hemicellulose	Lignin
PETG	0.39 ± 0.01	79.3 ± 2.37	0.62 ± 0.02	19.69 ± 0.59	70.59 ± 2.12	5.6 ± 0.17	0.5 ± 0.02	0.05 ± 0.00	23.76 ± 0.71	0.08			
OP	6.87 ± 0.21	67.12 ± 2.01	7.64 ± 0.23	18.37 ± 0.55	48.22 ± 1.45	8.05 ± 0.24	1.48 ± 0.04	0.66 ± 0.02	41.59 ± 1.25	0.17	23.3 ± 0.78	10.7 ± 0.32	27.5 ± 0.83

PETG, poly (ethylene terephthalate) glycol; OP, olive pomace; FC, fixed carbon

### Filament Qualitative Description and 3D Printing Process

Table 1 provides a detailed summary of the experimental conditions derived from the response surface design. This table is essential for understanding the influence of each factor on the final filament properties. Detailed analysis presented in Table 4 showed that studied variables (particle size, biomass content (v/v), and extrusion number) have a decisive effect on the filament phenotype. In this context, the extruder becomes a unit operation to produce composites, which is affected by various factors that can influence the manufacturing process, degrading its quality. Table 4 summarizes the characteristics from filaments produced based on the proposed response surface study.

**Table 4:**
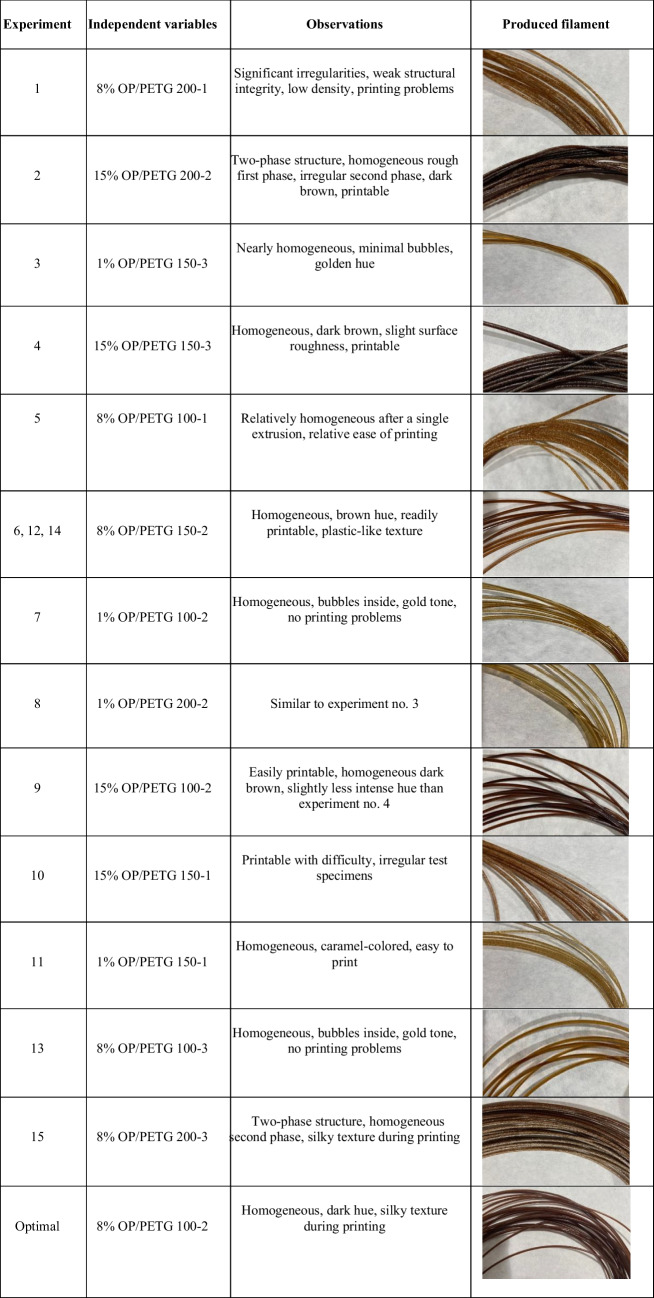
Phenotype of the filament based on visual observation and user experience during the printing of the test specimens

*OP*, olive pomace; *v*, volume; *PETG*, poly (ethylene terephthalate) glycol

It was shown that smaller particles (< 100 µm) improved filament homogeneity and printing capability. In contrast, larger particles (ranging 150–200 µm) led to more irregularities and less homogeneous structures, especially when combined with a reduced number of extrusions. This observed effect corresponds to processes occurring during compression, where the material is heated and melted, while being compressed by the screw thread (Hyvärinen et al. 2020). During this phase, the polymer moved along the screw helical channel, resisted by friction forces between solid and barrel, and between solid and screw surfaces. Increasing the particle size turned out in increasing resistance to movement, resulting in a torque that drove the solid plug forward, providing a more irregular volumetric flow. This effect is accompanied by poorer heat diffusion. Extrusion ended when the molten polymer was forced through the die, achieving the final filament diameter. At this point, to ensure continuous extrusion, the interaction between phases was critical. However, due to the characteristics of the materials used to improve homogeneity, other effects occurred, i.e., gas production due to poor compaction, along with a series of thermal reactions, resulting in high irregularity in the first extrusion process.

A clear relationship between number of extrusions, quantity of added biomass (OP), and particle size on filament quality (final material properties) for 3D printing is observed. A higher number of extrusions significantly improve filament homogeneity and stability. For example, in experiment no. 1, with a single extrusion and a particle size ranging from 150 to 200 µm, significant irregularities are observed. In contrast, experiment no. 4 (15% OP (v/v), three extrusions, and particle size ranged 100–150 µm and) resulted in a homogeneous, dark brown filament with slight roughness. Another notable case is provided by experiment no. 10, where a single extrusion, 15% OP (v/v), and 150–200 µm particle size were used. In this case, the filament showed low density and irregularities. In comparison, experiment no. 15 which used three extrusions, 150–200 µm particle size, and 8% OP (v/v) produced more homogeneous structure and uniform texture filaments. Results demonstrated that higher number of extrusions can improve filament quality, even with larger particle size.

However, extrusion process increased thermal aging, which can affect polymer properties. In the studied composites, it may be seen that it activates chemical processes that accelerate thermal oxidation processes, leading to chain scission and crosslinking in polymers. On a rheological level, the continued extrusion process can lead to variations in temperature-dependent viscosity, which can impact the extrusion process and lead to gel formation due to crosslinking.

Furthermore, in the lignocellulosic matrix, positive effects may be found. During extrusion, cellulose and lignocellulose may undergo thermal degradation. This results in decreased viscosity and improved flow properties, especially at high temperature and low humidity levels (Duque et al. 2017). Lignin, more thermally stable, softens at high temperature, favoring material flow similarly to that of biopolymers near their glass transition temperature, compared to pure cellulose or hemicellulose (Yang et al. 2019a, b; Ribca et al. 2021). Viscosity and flow behavior of these compounds during extrusion are influenced by interactions between cellulose, lignin, and other components, similarly to that of starch-based biopolymers. Therefore, the increase of biomass content in the mixture has also a significant impact on filament quality. Higher OP concentrations tend to produce denser material with darker hues, as may be seen in experiments with 15% OP (v/v). However, this can also lead to the formation of two-phase structures and increased surface roughness, negatively affecting printing quality. On the other hand, lower OP concentrations produce lighter filaments with fewer bubbles and lighter hues, such as gold or caramel, favoring smoother and more consistent printing. Mechanical properties and the impact of concentration on the quality of processed composites will be later discussed.

Regarding the thermal effect, on one hand, volatile formation alters filament structure, producing higher roughness and a significant impact on diameter homogeneity. On the other hand, polymer color is strongly affected by caramelization and Maillard reactions. Furthermore, it undergoes a noticeable shift from lighter hues (e.g., gold, caramel) to darker shades with increasing OP concentration. Maillard and caramelization reactions are favored by the presence of reducing sugars and amino acids in OP. As temperature increases during extrusion (typically around 200 °C), these compounds interact, leading to the formation of melanoidins (pigments responsible for the brown coloration) (Kathuria et al. 2023). The residence time of the polymer-biomass mixture within the extruder also plays a significant role, with longer residence times potentially enhancing Maillard and caramelization reactions. The extrusion process occurs at 200 °C and polymer/biomass mixture residence time ranges from 1 to 2 min. The extrusion process itself can be likened to a fast-pyrolysis effect, with moderate oxygen concentrations. This environment leads to the decomposition of the polymer and biomass. Thus, volatiles are generated, providing the necessary conditions for Maillard and caramelization reactions between N-groups and glycosidic groups to occur (Capone et al. 2007; Martínez-Bustos et al. 2012).

Printing capability is summarized in Table 4, where a clear distinction between filaments successfully printed and those that presented some printing difficulties is shown. Easy-to-print filaments, found in experiments no. 2, 4, 7, and 13, exhibited adequate quality due to a favorable combination of number of extrusions, biomass concentration (OP), and particle size. For example, experiment no. 2 (15% OP (v/v), 150–200 µm, and two extrusions) produced a two-phase structure filament, still printable with a homogeneous roughness. Similarly, experiment no. 13 (three extrusions, < 100 µm particle size, and 8% OP (v/v)) resulted in a homogeneous filament with a golden hue and no printing issues.

Although some filaments were difficult to be printed due to issues with homogeneity and density, in experiment no. 1 (single extrusion, 150–200 µm particle size, and 8% OP (v/v)), the filament showed significant irregularities, making it less appropriate for 3D printing. A similar case is seen in experiment no. 5 (< 100 µm particles with a single extrusion, 8% OP (v/v)) that resulted in a difficult-to-print material due to problems in the first extrusion.

**Fig. 3:**
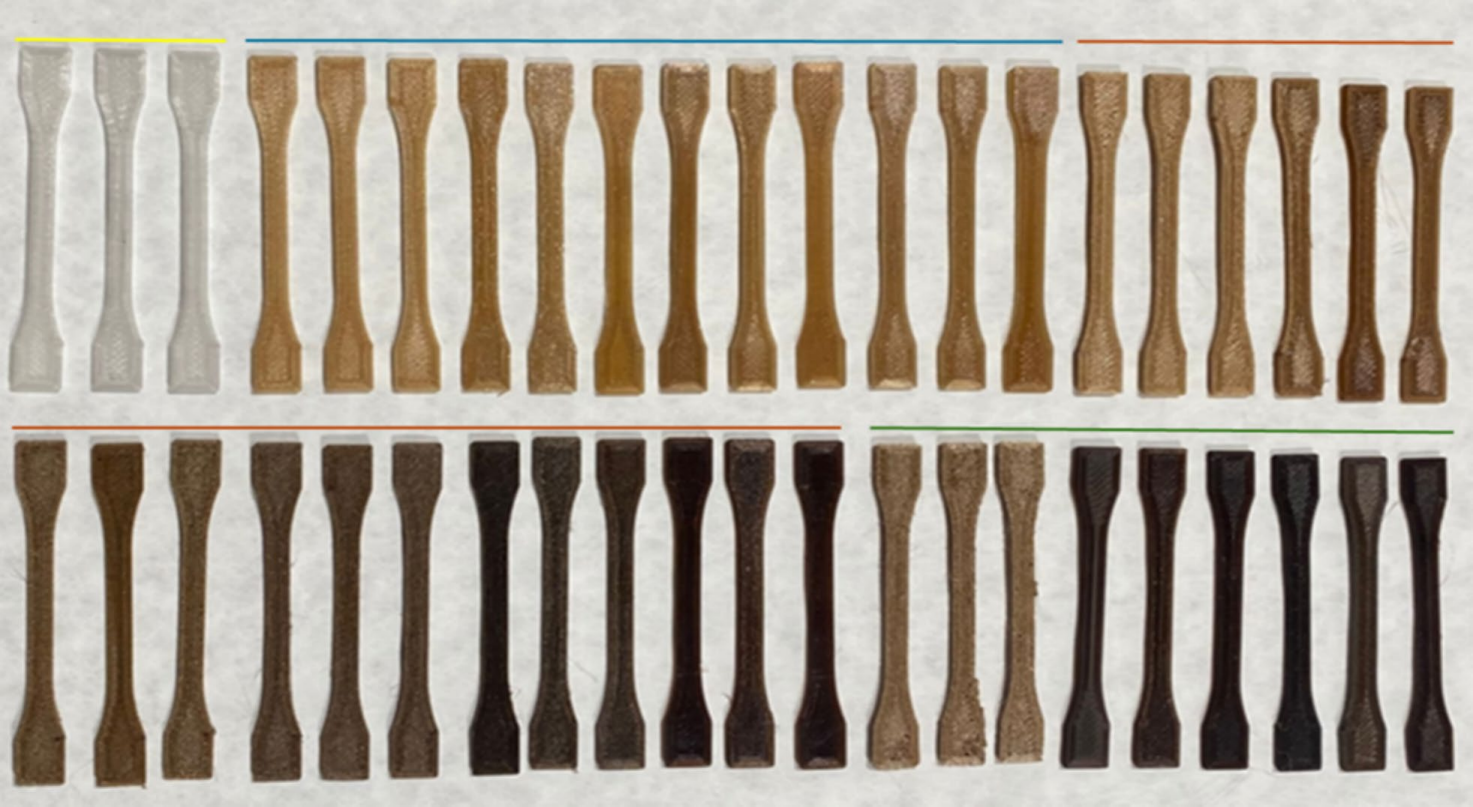
Manufactured test specimens ordered by OP content (0% yellow, 1% blue, 8% orange, 15% green), and number of extrusions (increasing from left to right within each percentage)

Figure 3 shows the resulting specimens, displaying the final range of colors after the printing process. This is one of the most interesting findings from the phenotype study. Thus, it highlights a significant aspect in the production of these composites: the use of OP as a coloring agent. Hues range from amber yellow to brown color, in a gradient that could be used in 3D printing processes for decorative purposes. Currently, natural pigments, such as spirulina, curcumin, beetroot, and chlorophyllin, are used in polymer bases, like PLA or polybutylene succinate (PBS), in concentrations ranging from 2 to 6% (w/w) (Yadav et al. 2023; Ibáñez-García et al. 2024). To evaluate their potential as value-added filaments, sample mechanical property studies were conducted.

**Fig. 4:**
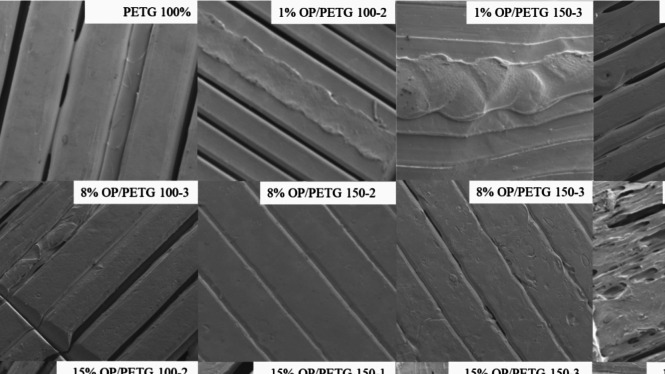
SEM images of surfaces of OP/PETG samples. Higher OP content and varying printing conditions increase porosity and interlayer separation

Figure 4 shows an enlarged view of the surface of the specimens, at 1 mm, where the final finish of the printed layers can be observed. This clarifies the impact of the selected parameters, specifically the extrusion number, to some extent and particle size on the surface morphology, while maintaining fixed the printing conditions. It is evident, from SEM images, that the number of extrusions exerts the most significant visual effect on the resolution and quality of the printed layers. A clear example of this degradation can be observed in samples 1% OP/PETG 150–3, 8% OP/PETG 150–3, and 15% OP/PETG 150–3. These samples, subjected to a higher number of extrusions, consistently present rougher surfaces, irregular layer deposition, visible cracks, processing artifacts, and deformations. This is attributed to a cumulative loss of flow continuity during repeated melting and solidification, compounded by heat diffusion issues, both within the nozzle and the polymer melt. Furthermore, interference with repolymerization processes and potential thermal degradation, due to multiple thermal cycles, contribute to mentioned surface defects. Ultimately, it compromises adhesion between layers and linearity of printed paths.

Within this context of higher extrusion number, specifically observed in the 8% OP/PETG series with 3 extrusions (8% OP/PETG 100–3, 8% OP/PETG 150–3, and 8% OP/PETG 200–3), a secondary effect related to particle size was also discerned. While all exhibit a general degradation associated with three extrusions, the sample with 150–200 µm particle size (8% OP/PETG 200–3) appears to show even more pronounced and severe surface irregularities, including larger voids and a more disrupted structure, compared to those with smaller particle sizes (< 100 µm and 100–150 µm) at the same extrusion level. This suggests that larger particle sizes, when combined with high extrusion number, further exacerbate processing challenges, potentially by impeding significantly flow or generating larger defects.

In contrast, these severe surface defects are markedly less pronounced, or even absent, in composites with particle sizes ranging between 100 and 150 µm and subjected to only two extrusions. This observation suggests a more robust processing window for these specific conditions, yielding a viability range for the bio-by-product between 8 and 15% (v/v).

While mechanical properties analysis identified particle size as a critical factor influencing the bulk material strength and ductility, SEM analysis of the printed surface highlights the paramount importance of the extrusion process on the esthetic and superficial integrity of the final part. The observed surface irregularities due to increased extrusions, i.e., cracks and poor layer adhesion, directly impact the visual quality. Moreover, they could serve as initiation site for defects, potentially affecting properties, like fatigue resistance. This distinction underscores that different characterization methods provide complementary insights into the complex interplay between material composition, processing parameters, and resulting composite properties. On one hand, the number of extrusions primarily influences processing stability and physical appearance of the printed part. On the other hand, particle size plays a more direct role in the internal stress transfer and overall mechanical performance of the bulk composite, with its impact on surface quality becoming more evident under severe processing conditions (e.g., high extrusion number).

### Tensile Test Results and Filament Optimization of OP/PETG Composites

Once the different filaments were produced and the corresponding composites were manufactured, to study the influence of each variable, mechanical properties were measured through surface response analysis. First, mechanical properties were examined. Then, to determine which mixture exhibited optimal behavior, a statistical analysis of OP/PETG mixtures was performed.

#### Mechanical Property Test

**Fig. 5:**
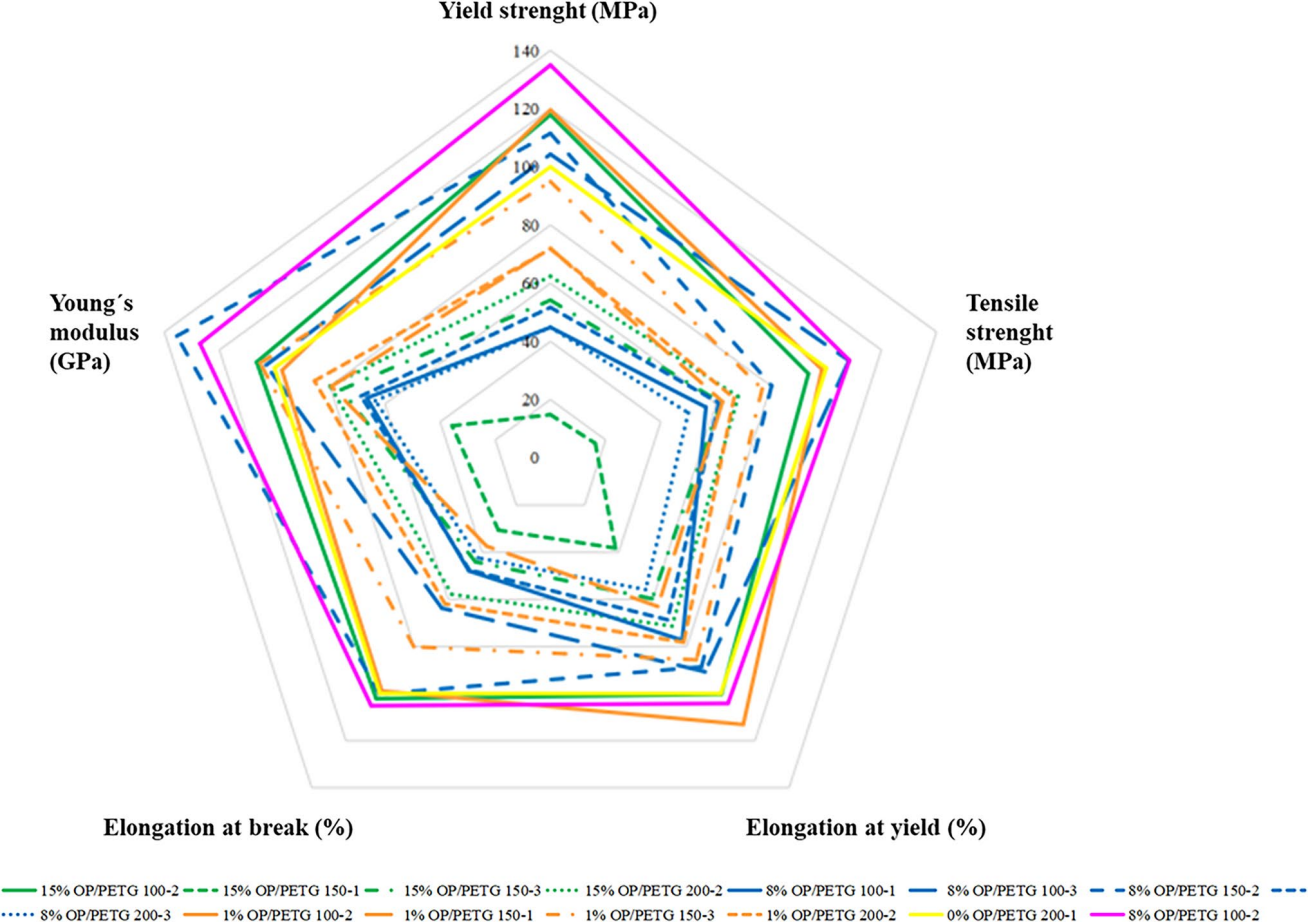
Radial plot analysis of OP impact on PETG mechanical behavior. Mechanical properties of the optimal OP/PETG filament composite. Each variable is represented by a different axis and the length of each axis segment corresponds to the magnitude of the corresponding property

Mechanical properties were related to tensile strength. This is due to the objective of evaluating composite resistance to stretching and their elongation capacity before failure. This will allow to carry out an analytical assessment of the response variables (particle size, OP/PETG (v/v) ratio and extrusions number). Results are shown in Table 5. Specimens from experiment no. 10 (high OP content and a single extrusion step) in Table 5 exhibited a higher dispersion in their mechanical properties. This variability is attributed to irregularities in interlayer adhesion and a non-homogeneous material distribution. This evidenced difficulties in the additive manufacturing process under these specific conditions. Sample comparison related to pure PETG specimens is found in Fig. 5.

**Table 5:** Tensile strength, failure strain, and Young’s modulus results of OP/PETG 3D-printed and their standard deviation

Experiment	TAG	Yield strength (MPa)	Tensile strength (MPa)	Elongation at yield (%)	Elongation at break (%)	Young’s modulus (GPa)
1	8% OP/PETG 200–1	17.98 ± 2.41	16.94 ± 2.13	2.30 ± 0.06	2.58 ± 0.68	1.27 ± 0.31
2	15% OP/PETG 200–2	21.70 ± 2.87	18.88 ± 2.59	2.39 ± 0.09	3.14 ± 0.73	1.47 ± 0.37
3	1% OP/PETG 150–3	33.02 ± 11.97	21.28 ± 8.76	2.87 ± 1.04	4.35 ± 2.44	1.94 ± 0.27
4	15% OP/PETG 150–3	18.84 ± 4.93	17.11 ± 4.41	2 ± 0.25	2.39 ± 0.62	1.40 ± 0.37
5	8% OP/PETG 100–1	15.60 ± 3.23	15.63 ± 2.36	2.59 ± 0.65	2.60 ± 0.69	1.23 ± 0.25
6	8% OP/PETG 150–2	38.78 ± 8.11	22.23 ± 2.54	2.97 ± 0.47	5.44 ± 1.77	2.49 ± 0.35
7	1% OP/PETG 100–2	41.58 ± 8.28	27.28 ± 10.69	3.79 ± 0.63	5.37 ± 1.72	1.79 ± 0.33
8	1% OP/PETG 200–2	24.96 ± 5.47	18.46 ± 4.08	2.62 ± 0.54	3.36 ± 0.85	1.58 ± 0.21
9	15% OP/PETG 100–2	41.01 ± 6.62	25.93 ± 8.55	3.36 ± 0.31	5.55 ± 1.17	1.96 ± 0.16
10	15% OP/PETG 150–1	5.16 ± 0.71	4.5 ± 0.96	1.28 ± 0.43	1.66 ± 1.16	0.66 ± 0.28
11	1% OP/PETG 150–1	25.07 ± 12.09	17.3 ± 11.18	2.11 ± 0.86	2.03 ± 1.04	1.46 ± 0.33
12	8% OP/PETG 150–2	35.15 ± 7.41	24.61 ± 2.68	2.58 ± 0.37	4.46 ± 1.43	2.25 ± 0.32
13	8% OP/PETG 100–3	36.29 ± 10.27	29.99 ± 9.54	3.04 ± 0.7	3.46 ± 0.99	1.90 ± 0.32
14	8% OP/PETG 150–2	44.23 ± 8.32	18.44 ± 2.13	3.25 ± 0.51	7.28 ± 1.83	2.25 ± 0.33
15	8% OP/PETG 200–3	15.59 ± 1.76	13.86 ± 1.92	1.87 ± 0.23	2.3 ± 0.56	1.17 ± 0.18
Blank	0% OP/PETG 1000–1	34.78 ± 10.88	27.72 ± 9.21	3.35 ± 0.89	5,43 ± 0.31	1.84 ± 0.32

*OP*, olive pomace; *PETG*, poly (ethylene terephthalate) glycol

Figure 5 shows an overview of the relationship between boundary conditions (OP (v/v) content (1%, 8%, 15%) added to PETG as filler, particle size (100, 150, 200 µm), and extrusion number (1, 2, 3)) and mechanical properties (yield strength (MPa), tensile strength (MPa), elongation at yield (%), elongation at break (%), and Young’s modulus (GPa)) of the resulting OP/PETG specimens.

Figure 5 compares mechanical properties of different composites with the control sample, pure PETG (pink line), whose properties were considered a reference and evaluated for comparison purposes. The most informative value is the Young’s modulus, which helps understanding sample degree of rigidity. It also provides information on the stress that composites can withstand before undergoing permanent deformation. In the series of conducted experiments, a maximum of 2.5 GPa was observed in experiments no. 6, 12, and 14 (DoE central points), containing 8% OP (v/v) and a particle size ranged 100–150 µm. Figure 5 shows that all these samples have a higher Young’s modulus (about 1.4 times above that of pure PETG). It also illustrates that 15% OP (v/v) with < 100 µm particle size yields similar modulus values. Regarding the number of extrusions (which implies a higher thermal footprint), it was observed that two extrusions provided the optimal value. Vijayasankar et al. (2022) used silk as natural fiber and reported a modulus 2.5 times higher than PETG, with a maximum concentration of 10% (w/w), similar to this study (Vijayasankar et al. 2022). The study demonstrated that short silk fibers help supporting high load before fracture. In their study, tensile strength showed values above 20 MPa, while the maximum value from this study was 29.99 MPa (experiment no. 13, 8% OP (v/v), and < 100 µm), and the minimum 4.5 (experiment no. 10, 15% OP (v/v), maximum concentration). The higher value represents high tensile strength, allowing the material to withstand higher stretching forces without breaking. In contrast, the lower value indicates low tensile strength and ease of the material to break with little stretching forces. However, adding biofibers does not always mean an increase in mechanical properties. The synthesized reactive thermoplastic starch blend had lower, but acceptable, mechanical properties. However, adding 30% thermoplastic starch, 15% reduction in tensile strength, and 40% in deformation and impact resistance was also found (Kulkarni and Narayan 2023).

In the analysis of elongation at break (%), in most experiments, a decrease compared to the virgin material (0% OP/PETG 200–1) was observed. For instance, experiment no. 11 (15% OP/PETG 150–1) exhibited a significant reduction of 69.42%, indicating that this material is less ductile and breaks more easily under tension. In contrast, 15% OP/PETG 100–2 shows a slight improvement of 2.21%, suggesting that this material retains elongation comparable to the virgin material, being slightly more resistant. These variations in elongation reflect the influence of both composition and treatments over material ability to deform before breaking. Moreover, this value increases with the number of extrusions (up to two). Exception is provided by 3 extrusions that slightly decreases the value. These variations will be studied in detail in the following section.

The value representing high tensile strength allows the material to withstand higher stretching forces without breaking. Several authors have reported an increase in this value, from 31.36 to 41.62 MPa (a 32.80% increase) (Huang et al. 2018). This was achieved by modifying extrusion temperatures (with an optimal value of 190 °C) and through the chemical and physical treatment of biofibers. In the present study, as shown in Fig. 4, best results were provided with 8% OP (v/v); the higher the number of extrusions the higher the parameter improvement. The maximum value was provided by experiment no. 13 (8% OP/PETG 100–3) with a value of 29.99 MPa, representing an 8% increase compared to pure PETG. The minimum was 4.5 MPa, from experiment no. 10 (15% OP/PETG 150–1).

**Fig. 6:**
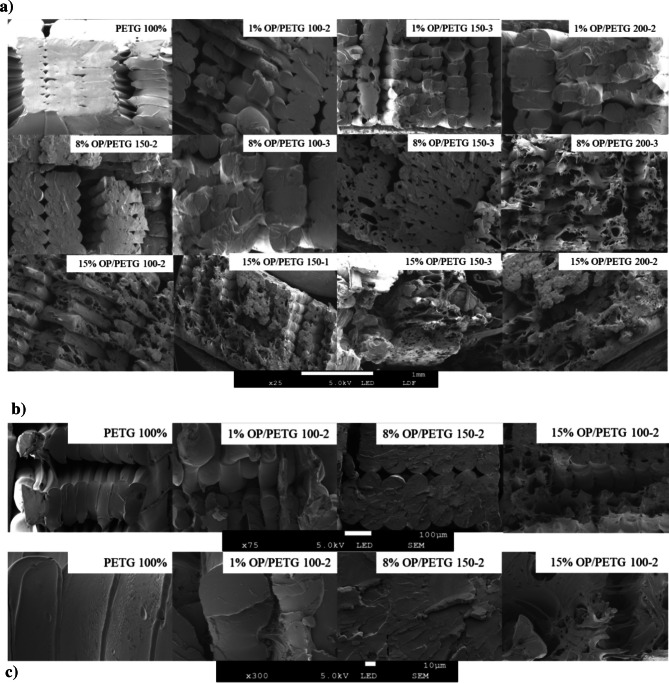
SEM images of fracture cross-sections of OP/PETG composites at varying magnifications: a × 25 magnification (scale bar 1 mm), overall view of layer adhesion and fracture paths; b × 75 magnification (scale bar 100 µm), intermediate view of internal structure and particle distribution for selected samples; c × 300 magnification (scale bar 10 µm), high-magnification view of matrix-particle interface and micro-fracture details for selected samples

To complete the analysis of mechanical properties, study of variable behavior, and understand the underlying causes leading to optimization, Fig. 6 presents SEM images of the fracture cross-sections of tested specimens. Images clearly show the relationship between particle size, number of extrusion cycles, and amount of reinforcing material.

The incorporation of biofibers, i.e., OP, into PETG matrices can significantly impact the mechanical integrity of the composite material. These effects have been reported in previous studies, using ABS (Carmona-Cabello et al. 2025) or olive stone powder (Burgos Pintos et al. 2024). The observed enhancements in mechanical properties of OP/PETG composites may be attributed primarily to a filler/stiffening effect, where the rigidity of OP particles restrict the mobility of PETG polymer chains, leading to increased stiffness and strength. This is notably influenced by OP particle size, with smaller particles (< 100 µm) facilitating more uniform dispersion and a higher interfacial area for efficient stress transfer from the PETG matrix to the filler. Furthermore, optimized extrusion cycles (specifically, two extrusions) likely contributed to improved particle dispersion and enhanced matrix-filler interfacial adhesion through wetting, promoting better physical interlocking and load distribution. A nucleation effect by OP particles on PETG matrix may also contribute to increased rigidity.

However, the presence of non-structural macromolecules, i.e., proteins, waxes, and hydrated carbon, within OP may reduce interfacial adhesion between fibers and matrix. Additionally, internal moisture evaporation and thermal degradation during the extrusion process (at 200 °C) may lead to voids formation. These issues are visually evident in fracture cross-section images (Fig. 6), which shows a progression from pure PETG ductile fracture to rougher and more porous surfaces with increasing OP concentrations (1%, 8%, 15% (v/v)).

While an optimal 8% OP (v/v) content may allow higher energy dissipation by promoting good dispersion and adhesion, higher load, i.e., 15% OP (v/v), often shows significant porosity and structural degradation, indicating poorer fiber dispersion and weak matrix adhesion. However, the feasibility of incorporating up to 15% OP without completely detrimental effects highlights its potential for higher plastic reduction. Additionally, particle size (< 100, 100–150, 150–200 µm) and number of extrusion cycles (1, 2, 3) strongly influence composite morphology; larger particle size and repeated extrusions contribute to more severe fragmentation and porosity. Magnified images (Figs. 6b and 6c) confirm a loss of surface homogeneity and increased roughness, indicating reduced biofiber integration, consistent with similar findings where increased additive loading leads to artifacts or voids associated with fracture brittleness (Petousis et al. 2024a).

#### Mechanical Properties of Optimal OP/PETG Filament Composite

Once all tests and analysis were finished, a Box-Behnken regression analysis was performed, providing a quadratic polynomial regression model for each mechanical property (Table 6). To identify the key parameter that exerts a significant influence on OP/PETG blend mechanical properties, three-way ANOVA with interaction of all factors were analyzed (Table 6). As may be seen, OP particle size emerged as the most influential variable among all studied mechanical properties.

**Table 6:** Regression equations based on ANOVA for the most influential variables for each mechanical property

Mechanical property response	Predictive equations	*R*^2^	Controlling variable	(*p*-value < 0.05)
Yield strength (MPa) =	2.9 + 2.22X – 0.003Y + 42.9Z—0.1122XX – 0.000388YY – 9.34ZZ—0.00192XY – 0.284XZ- 0.0235YZ (1)	0.939	Particle size OP (%)* OP (%) Extrusion number * Extrusion number	0.001 0.042 0.006
Tensile strength (MPa) =	41.2 + 0.616X – 0.351Y + 13.24Z – 0.0385XX + 0.000917YY – 2.14ZZ + 0.00126XY – 0.142XZ – 0.0217YZ (2)	0.928	Particle size	0.001
Elongation at yield (%) =	0.321–0.0026X – 0.00887Y + 3.953Z + 0.00038XX + 0.000021YY – 0.7437ZZ + 0.000143XY – 0.02714XZ – 0.00385YZ (3)	0.989	Particle size OP (%) Extrusions number Extrusions number * Extrusions number Particle size * Extrusions number Extrusions number * OP (%)	0.001 0.003 0.004 0.001 0.014 0.015
Elongation at break (%) =	8.81 + 0.369X + 0.0538Y + 9.71Z- 0.01184XX – 0.000202YY – 2.070ZZ- 0.000286XY – 0.0829XZ – 0.00310YZ (4)	0.928	Particle size Extrusions number * Extrusions number	0.017 0.001
Young modulus (MPa) =	− 3975 + 178.1X + 47.3Y + 2559Z – 7.78XX – 0.1652YY – 564.7ZZ – 0.196XY—17.0XZ – 0.65YZ (5)	0.956	Particle size Particle size * Particle size OP (%) * OP (%) Extrusions number * Extrusions number	0.006 0.004 0.006 0.001

*X*, OP (volume fraction, %) in the blends; *Y*, particle size (µm); *Z*, extrusion number; *OP*, olive pomace

Smaller particles with larger surface area for interfacial interactions can contribute to better reinforcement and improved mechanical performance (Siddikur Rahman et al. 2018). In addition to OP concentration, the effect of particle size also shows an impact on the properties. Specimens with smaller particle size generally exhibited higher yield strength, tensile strength, and Young’s modulus. Moreover, smaller particles provide a larger interfacial area for adhesion between filler and matrix, leading to improved stress transfer and better mechanical performance.

Proposed quadratic polynomial regression models include linear, quadratic, and interaction terms between variables. Equation 1 in Table 6 represents the adjustment equation for yield strength. The equation shows that yield strength increases with OP content and the number of extrusions, while OP particle size has a minor negative effect.

Similar to yield strength, tensile strength (Eq. 2) also increases with OP content (%) and number of extrusions, while particle size has a negative effect. This aligns with the observation that smaller particles improve tensile strength, due to better stress distribution and a larger interfacial area for adhesion. Elongation at yield (Eq. 3) is negatively affected by OP content (%) and particle size, but positively by the number of extrusions. The equation for elongation at break (Eq. 4) shows a complex relationship where OP content (%), particle size, and number of extrusions play important roles. The equation of Young’s modulus (Eq. 5) suggests that material becomes stiffer with a higher OP content (%), smaller particle size, and increase of extrusion number.

**Fig. 7:**
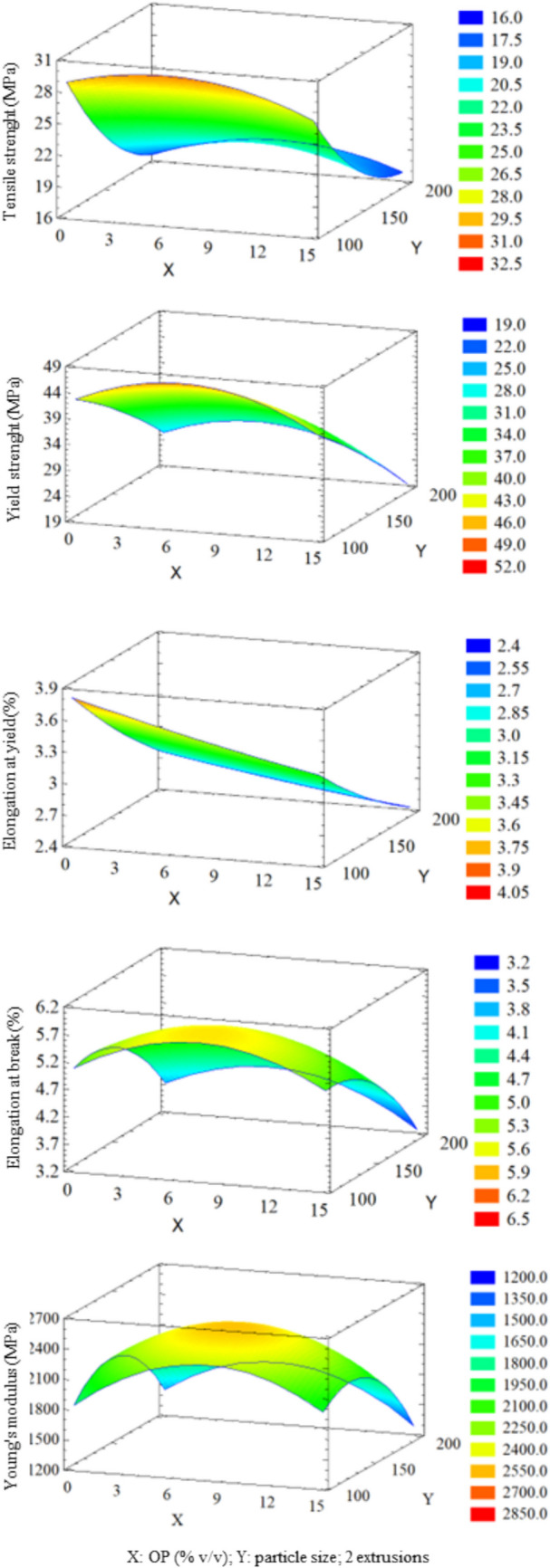

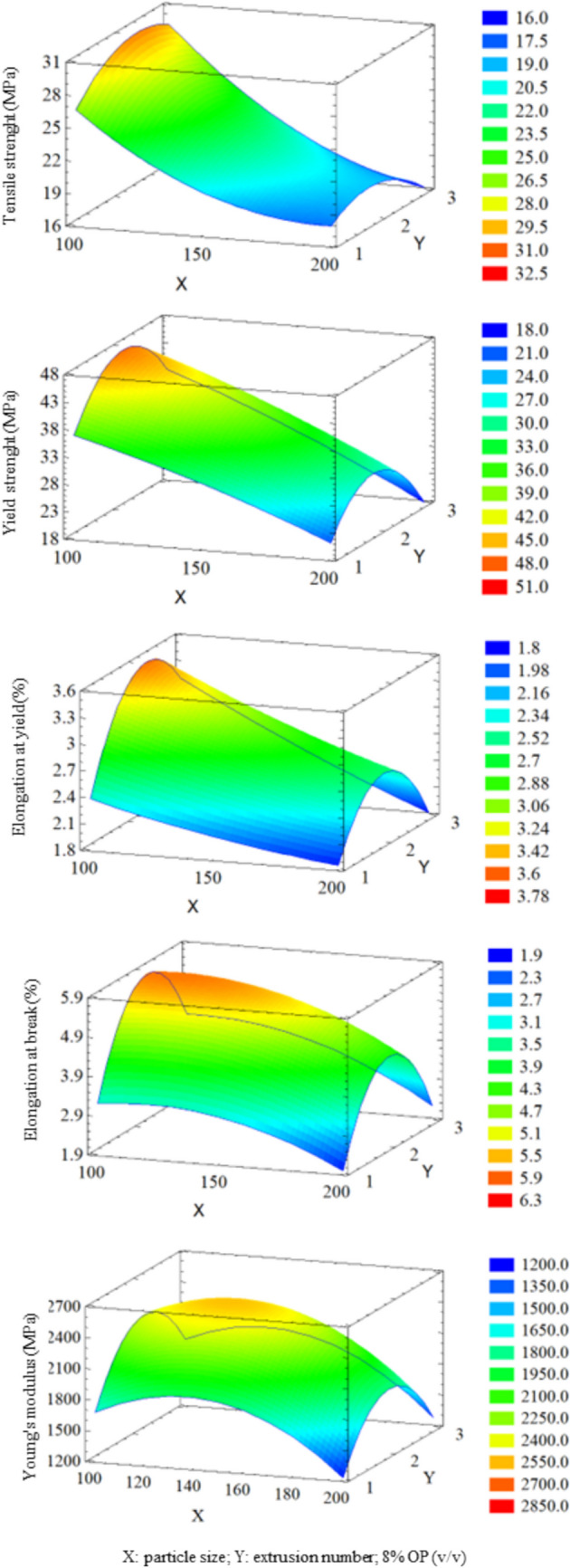

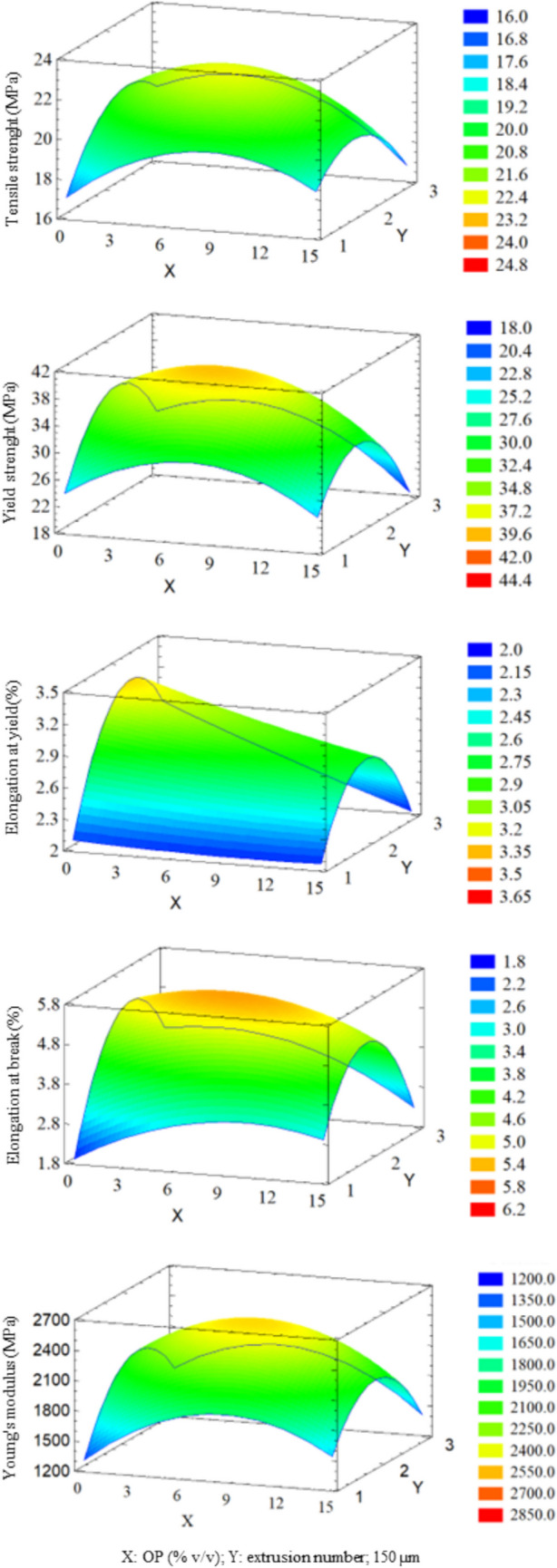
3D graphical representation of response surface methodology results. *X* and *Y* axes represent two independent variables, while *Z* axis represents each specific mechanical property

Thus, once tests and analysis were finished, to optimize the mechanical properties, a RSM was carried out. Figure 7 summarizes the surface response plots that visually represent the interaction effects between control variables and resulting mechanical properties. In each plot, *X* and *Y* axes represent two independent variables, while *Z* axis represents each specific mechanical property. To examine the interplay between the two independent variables and the mechanical property, the value of the third independent variable was fixed at its intermediate level. This approach allows for a focused analysis of the influence of the two controlling variables on the mechanical property under consideration.

As can be seen in Fig. 7, the addition of OP as a filler to PETG composites generally had a positive impact on the material mechanical properties. This is primarily attributed to the reinforcement effect of OP particles, which distribute stress throughout the PETG matrix, enhancing its resistance to deformation and failure. Yield strength values ranged between 20 and 45 MPa. According to ANOVA (Table 6), OP particle size was the most significant variable. Smaller particle sizes were associated with higher yield strength. OP content (%) and the number of extrusions also affected yield strength, although the relationship is not linear. In this sense, maximum yield strength was achieved when 8% OP (v/v) is added and two extrusions are performed. Tensile strength values ranged between 18 and 30 MPa. Experimental results showed that smaller OP particle sizes are linked to higher tensile strengths.

Elongation at yield values ranged between 2 and 3.5%. As reported by ANOVA (Table 6), several variables and their interactions influenced this property. This included OP particle size, OP content (%), number of extrusions, interaction between number of extrusions, particle size and number of extrusions, and OP content (%) and number of extrusions. The highest elongation at yield was achieved with small particle size, low OP content (%), and two extrusions. Elongation at break values ranged between 2 and 6%. Unlike elongation at yield, elongation at break was influenced exclusively by OP particle size and the interaction between the number of extrusions (Table 6). Smaller OP particle sizes and a second extrusion were associated with higher elongation at break. Increased elongation resulted in a larger area under the curve, indicating a tougher material.

Young’s modulus values ranged between 1500 and 2500 MPa. According to Table 6, the most influential variables were OP particle size, interaction between OP particle sizes, interaction between number of extrusions, and interaction between OP content (%). Experimental results showed that an 8% OP (v/v), two extrusions, and a medium-to-small OP particle size yielded the highest Young’s modulus, resulting in a stiffer material.

Results from Table 6 and Fig. 7 show that OP particle size emerged as the most recurrent and influential variable, exhibiting the lowest *p*-value. In general, smaller particle sizes are correlated with higher values of the output variables, resulting in stiffer, stronger, and tougher materials. The number of extrusions was the second most significant variable, impacting both linearly and quadratically. This variable proved to be critical for material performance. In fact, a marked improvement in material properties from the first to the second extrusion, followed by a decline with a third extrusion, was demonstrated. Also, OP content (%) used as filler significantly influenced material properties.

According to Fig. 7, the optimal values to enhance material mechanical properties were 8% OP (v/v), 100 µm particle size, and two extrusions. The filament produced under these experimental conditions is shown in Table 4. While the filament produced under the conditions of experiment no. 12 (8% OP/PETG 150–2) showed a 35% increase in Young’s modulus, optimal conditions were chosen as a compromise solution to achieve balanced enhancements among all mechanical properties.

The mechanical properties of the specimen produced with the filament under optimal experimental conditions demonstrated notable improvements: 34.99% increase in yield strength, 8.41% in tensile strength, 4.18% in elongation at yield, 5.16% in elongation at break, and 27.05% in Young’s modulus (Fig. 5). Considering these outstanding results, this study presents a compelling case for the use of this optimized filament in applications demanding superior mechanical performance. Moreover, it offers a balanced enhancement in yield strength, tensile strength, elongation, and rigidity.

Numerous studies have explored PETG mechanical properties and sustainability in material extrusion (MEX). For instance, Vidakis et al. (2020) investigated the strain rate sensitivity of FFF-processed PETG, reporting significant variations of mechanical properties with loading speed and strain rate. Complementarily, Vidakis et al. (2021) highlighted PETG capacity for multiple recycling cycles, demonstrating its ability to largely keep acceptable mechanical properties even after reprocessing, thus supporting circular economy principles. In contrast, the present study focuses on enhancing virgin PETG through the incorporation of OP. Under optimized conditions (e.g., 8% OP content (v/v), < 100 µm particle size, and two extrusion cycles), formulations significantly improve mechanical performance, with Young’s modulus values reaching approximately 2.5 GPa (nearly double the lower range for pure PETG) and a maximum tensile strength of 29.99 MPa. While bio-composite elongation at break often decreases, optimal OP formulations kept or slightly improve elongation compared to virgin PETG. This indicates that certain formulations with OP can enhance stiffness and strength while retaining ductility. These findings underscore the potential of bio-based reinforcement additives for sustainable MEX materials that are suitable for applications requiring balanced mechanical performance. Furthermore, while an optimal OP loading of approximately 8% (v/v) provides peak performance, results demonstrate the feasibility of incorporating up to 15% (v/v) OP into the PETG matrix without detrimental mechanical effects. This higher achievable filler content significantly amplifies the potential for reducing petroleum-derived plastic use in sustainable additive manufacturing applications.

### Thermal Behavior of OP/PETG Filaments

**Fig. 8:**
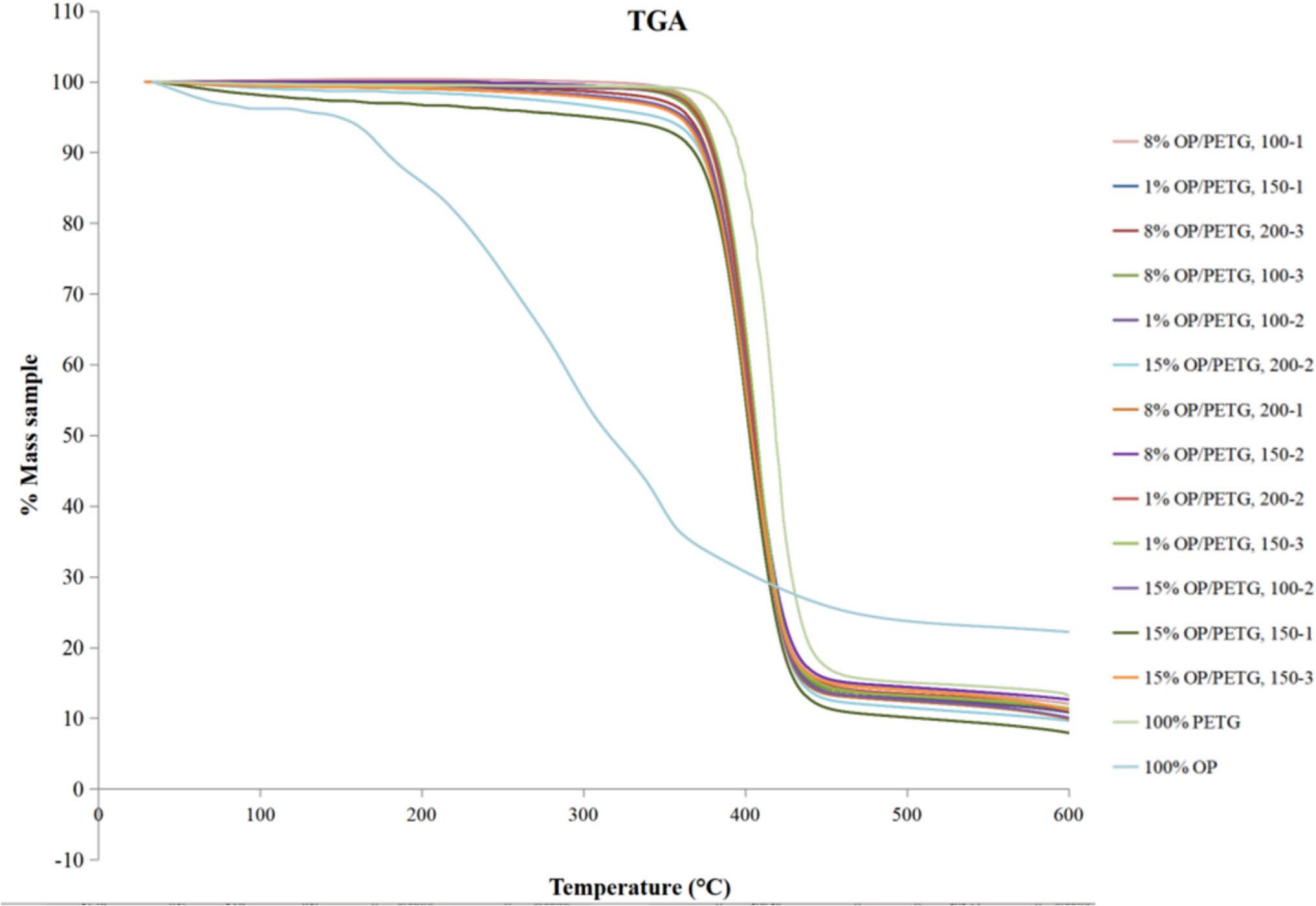
Thermogravimetric analysis of OP/PETG filaments

Thermal stability of OP/PETG specimens was studied by TGA. Thermogravimetric analysis showed that composites were thermally stable up to 300 °C. Thus, they could be used for melt extrusion process manufacturing. The thermogravimetric decomposition curves of the different blends are shown Fig. 8. As can be observed, the curves followed a similar trend, suggesting comparable thermal stability and decomposition mechanisms. This shared behavior could be attributed to the inherent characteristics of the polymer matrix, which dominates the overall degradation process. Despite the observed similarities, a key distinction lied in the initial degradation temperature. Table 7 presents the values of temperature corresponding to 5% and 95% mass loss, as well as the percentage of residual mass remaining at 600 °C. For compounds with higher OP content (%), the onset of degradation occurred at slightly lower temperatures, compared to neat PETG. This suggests that the presence of OP may introduce subtle changes in the structure or interactions of the polymer, resulting in a marginal decrease in thermal resistance. Analysis revealed that OP content (%) exerts a more noticeable influence on the reduction of initial degradation temperature (T_5%_). This suggests a decrease in thermal stability as the content of waste pomace increases. The number of extrusions seems to have a less consistent effect on thermal stability. Some cases showed a slight increase in T_5%_ with higher number of extrusions. This could indicate an improvement in the compatibility between PETG matrix and biomass particles. However, a clear trend cannot be elucidated from results. The number of extrusions also exhibits some influence, although to a lesser extent. At 600 °C, residual mass slightly increased with the number of extrusions. The remaining residue represents the fraction of the composite that was able to withstand high temperature without completely decomposing. Figure 8 suggests that OP particle size exerts a less pronounced influence on thermal stability compared to OP content and number of extrusions. Variations in mass loss between samples with different particle size, same OP content (%), and number of extrusions are minimal.

**Table 7:** Thermogravimetric analysis temperature values at 5% and 95% mass loss

Experiment	TAG	T_5%_	T_95%_	T_g_	Residual mass (%) at 600 °C
1	8% OP/PETG 200–1	361.58	663.41	78.50	9.87
2	15% OP/PETG 200–2	345.75	721.90	74.97	9.62
3	1% OP/PETG 150–3	377.33	759.49	79.29	12.30
4	15% OP/PETG 150–3	369.53	745.92	74.93	14.47
5	8% OP/PETG 100–1	374.42	731.00	82.45	11.98
6, 12, 14	8% OP/PETG 150–2	373.45	753.21	81.00	12.64
7	1% OP/PETG 100–2	377.57	742.88	83.51	10.80
8	1% OP/PETG 200–2	378.17	764.47	75.24	12.95
9	15% OP/PETG 100–2	366.16	734.76	76.82	11.11
10	15% OP/PETG 150–1	305.74	677.89	79.73	7.94
11	1% OP/PETG 150–1	378.66	758.12	81.00	13.38
13	8% OP/PETG 100–3	377.71	759.13	82.76	13.61
15	8% OP/PETG 200–3	374.14	744.86	77–00	14.13
OP	100% OP/150–0	145.57	715.65	-	22.16
Blank	0% OP/PETG 1000–1	387.93	742.46	83.00	9.24

*OP*, olive pomace; *PETG*, poly (ethylene terephthalate) glycol; *T*_*95%*_, temperature value at 5% mass loss; *T*_*5%*_, temperature value at 95% mass loss; *T*_*g*_, glass transition temperature

Comparing temperatures at 5% and 95% of mass loss, there was a clear trend according to the amount of biomass included in the final composite. Thus, on average, those temperatures tend to decrease, for T_5%_, from 377.93 to 346.80 °C (from 1 to 15%, v/v), and from 756.24 to 720.12 °C, for T_95%_, when the volume increased from 1 to 15%. This is expected due to the degradation behavior of OP, which is faster and follows a different pattern than neat polymer. Dehydration and depolymerization of cellulose processes occurred in the range of 150–300 °C. Lignin alkyl chains conversion was assigned to degradation up to 400 °C. From this temperature to the maximum measured one, charring reactions and aromatic chains conversion on lignin fraction can be found (Vuppaladadiyam et al. 2023). Likewise, this trend was also supported by the residual mass observed at 600 °C, which is slightly higher in the case of higher biomass volume fraction (13.61–14.13–14.47%).

**Fig. 9:**
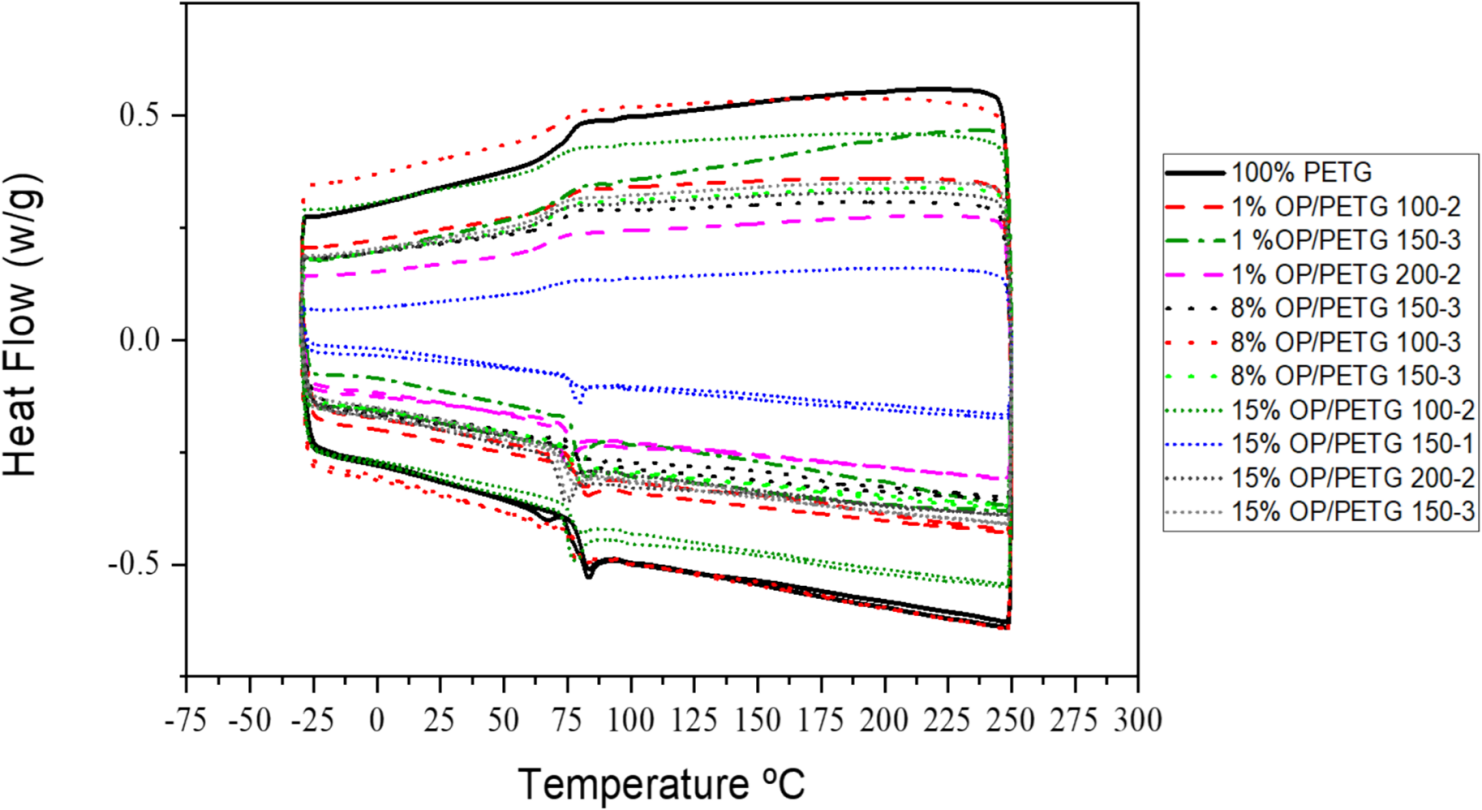
DSC analysis of OP/PETG filaments

Figure 9 shows DSC curves. As may be seen, pure PETG glass transition occurs sharply and narrowly, which is characteristic of homogeneous materials with uniform molecular weight distribution and no additives that alter its molecular mobility. As OP is incorporated, especially at higher concentrations (8% and 15% OP (v/v), glass transition temperature (T_g_) not only shifts to lower temperatures, but the transition also becomes broader and less defined. This broadening of the thermal event indicates higher structural heterogeneity in blends, due to local differences in composition, compatibility, and filler distribution.

The downward shift of T_g_ is a classic effect when less rigid phases or plasticizers are introduced into a polymer matrix. In this case, OP (being an organic residue with low molecular weight components and possibly lacking strong interaction with PETG) acts as a partial plasticizer, allowing chain mobility and, thereby, reducing T_g_. This effect is more pronounced with larger particles (ranging from 150 to 200 µm) or higher number of extrusions. It likely introduces defects or thermal degradation, reducing molecular weight and increasing polymer internal mobility.

On the other hand, the broadening of T_g_ reflects that different material regions have varying levels of thermal mobility. This may be due to non-uniform dispersion of OP or areas with different interactions between matrix and filler, providing microenvironments with varying chain mobility restrictions. This phenomenon can compromise material thermal stability, but also indicates a multiphase or structurally complex behavior, as is common in biocomposites with non-functionalized organic fillers. In sum, both downward shift and T_g_ broadening reveal a loss of thermal homogeneity caused by the addition and processing of OP.

### Infrared Spectral Analysis

**Fig. 10:**
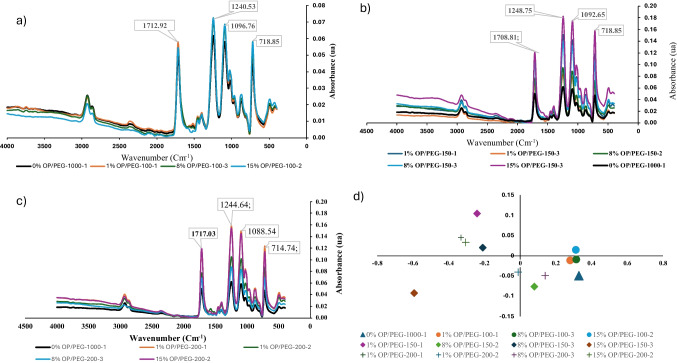
FTIR spectral analysis of pure PETG and OP/PETG composites

The infrared analysis was used as quick analysis of the quality of the homogenization and to correlate spectral data with physical properties and thermal decomposition characteristics. In Fig. 10, the different spectra can be observed, with the spectrum obtained from pure PETG (black line) included as a reference in all cases. The comparison was made by particle size: (a) < 100 µm, (b) 100–150 µm, and (c) 150–200 µm. To complete the study, a principal component analysis (PCA, Fig. 10d) was conducted. To perform a more exhaustive analysis, four base peaks were established: 1712.2 cm^−1^ as a reference for C = O stretching (ester groups), 1248.7 cm^−1^ for C(O)–O stretching, 2920.59 cm^−1^ for C-H stretching as a reference for aliphatic groups, and 872.60 cm^−1^ for out-of-plane C-H bending in aromatic rings (Li et al. 2023).

In an initial analysis, no significant variation in the spectral peaks was observed, although there was an increase in intensity as particle concentration and size increased. This is consistent with the literature. In a previous work, the introduction of pineapple fiber did not lead to the appearance of new bands, but did cause a reduction in their amplitude and an increase in their intensity (Kumar et al. 2024). Similarly, the appearance of new peaks was observed when other types of materials, such as activated carbon, were integrated (Bedi et al. 2023). Results shown an increase in intensity proportional to particle size, which reinforced the surface response analysis findings, highlighting the significant effect of particle size. This means an impact on extrusion and printing processes. PCA highlighted significant variations in FTIR spectra, particularly in C = O and C(O)–O stretching regions. Samples with larger particle sizes (100–150 and 150–200 µm) exhibited notable red shifts in these regions, as seen in the shifts to 1712.9 cm^−1^ and 1240.53 cm^−1^. These shifts suggest stronger interactions between carbonyl and ester groups with the polymer matrix. This is likely due to increased interaction with additives or structural modifications within the PETG.

In contrast, C-H stretching region only showed slight blue shifts, such as the shifts to 2923.48 cm^−1^ and 2924.18 cm^−1^. This indicates minor changes in the aliphatic structure, likely due to subtle alterations in the polymer chain from extrusion. The out-of-plane C-H bending peaks remained remarkably stable across all samples. This suggests that the aromatic structures within PETG are largely unaffected by changes in particle size or extrusion number. This stability is essential for preserving the composite structural integrity, even as other parts of the polymer undergo modifications.

On the other hand, notable variations could also be observed in 1455.04 cm^−1^ peak (asymmetric C-H bending in CH_2_). From there, moderate shifts and peak broadening suggest interactions with other matrix components, potentially due to additives or thermal modifications. The 1088.54 cm^−1^ peak (C–O–C stretching in ether) remained stable, indicating that the ether group is not significantly affected by processing. However, the 1392.52 cm^−1^ peak (C-H stretching in CH_3_) showed slight shifts and intensity changes. This suggested interactions between methyl groups and the matrix, possibly influenced by lignocellulose or thermal alterations. Other peaks, such as 1014.61 cm^−1^ (in-plane C-H bending in aromatic rings) and 718.85 cm^−1^ (out-of-plane C-H bending in aromatic rings), show varying degrees of stability and interaction. This highlights the influence of thermal modifications and matrix interactions. Peaks at 484.71 cm^−1^ (metallic/inorganic bond vibrations) and 2074.40 cm^−1^ (C≡C stretching in alkynes) exhibited stability. However, the 958.22 cm^−1^ peak (C-H stretching in benzene rings) indicated changes in configuration or exposure due to extrusion or additive incorporation (Bedi et al. 2023).

## Conclusions

This research aimed to enhance PETG filament for additive manufacturing via material extrusion by incorporating olive pomace (OP) filler. Our findings demonstrated that the extrusion process, a critical step in 3D printing, significantly influences mechanical properties and esthetic appeal of the composite. By carefully controlling factors, such as particle size, extrusion temperature, and OP concentration, a substantial enhancement in the material strength, stiffness, and toughness was achieved. Results indicated that particle size was the most influential parameter, with smaller particles leading to superior mechanical properties, such as stiffness, strength, and toughness.

Moreover, the incorporation of OP introduced a natural color variation, ranging from light beige to dark brown. This esthetic versatility expands the potential applications of the composite, making it suitable for a wider range of products, including those prioritizing visual appeal and sustainability.

While the study successfully demonstrated the potential of OP/PETG composites, further research is needed to fully optimize the processing parameters, addressing challenges related to viscosity and melt flow. Nevertheless, this work provides a strong foundation for the development of high performance, sustainable, and visually appealing 3D printing materials.

In the context of circular economy, this study focusses on applying the OP/PETG blend composite material in the production of components and spare parts for olive oil packaging lines. This innovative composite material, which combines PETG and OP, may contribute to a more sustainable and environmentally friendly approach. This valorization of OP directly reduces the reliance on fossil-based polymers, offering significant environmental benefits, especially in large-scale applications. This practice aligns with the principles of circular economy, as it involves reusing materials that, otherwise, would go to waste, thereby closing the loop and reducing the environmental impact.

## Data Availability

Data could be shared upon request.

## Abbreviations

3D: Three-dimensional
ABS: Acrylonitrile butadiene styrene
ANOVA: Analysis of variance
ATR: Attenuated total reflectance
DSC: Differential scanning calorimetry
DoE: Design of experiment
DT: Detrending
FC: Fixed carbon
FDA: Food and drug administration
FFF: Filament fused fabrication
FTIR: Fourier-transform infrared spectroscopy
GDP: Gross domestic product
LLDPE: Linear low density polyetyilene
MEX: Material extrusion
OP: Olive pomace
PA6: Polyamide 6
PBS: Polybutylene succinate
PCA: Principal component analysis
PET: Polyethylene terephthalate
PETG: Poly (ethylene terephthalate) glycol
PLA: Polylactic acid
PP: Polypropilene
PVC: Polyvinyl chloride
RSM: Response surface methodology
SEM: Scanning electron microscope
SG: Savitzky-Golay
SNV: Standard normal variate
T_5%_: Temperature value at 95% mass loss
T_95%_: Temperature value at 5% mass loss
T_g_: Glass transition temperature
TGA: Thermogravimetric analysis
UV: Ultraviolet
v: Volume
w: Weight
